# Special Issue: Multifunctional Cementitious Composites: Manufacturing and Characterization

**DOI:** 10.3390/ma18091976

**Published:** 2025-04-26

**Authors:** Hongzhi Zhang

**Affiliations:** School of Qilu Transportation, Shandong University, Jinan 250002, China; hzzhang@sdu.edu.cn

##  

The development of multifunctional cementitious composites has been a topic of interest in the construction industry in recent decades. Techniques developed in various fields have been implemented to create multifunctional cementitious composites with properties beyond what is possible with conventional technologies. For example, technological developments in additive manufacturing and nanotechnologies enable us to increase our efforts to develop better, stronger, and more optimal cementitious composites. These high-performance multifunctional cementitious composites, which have excellent mechanical properties and durability and the functions needed for building structures, are promising materials for implementing the sustainable, durable, and multifunctional development of infrastructures such as bridges, tunnels, dams, and nuclear power plants.

This Special Issue aims to provide readers with an overview of the current research progress on the manufacturing and characterization of multifunctional cementitious composites. It includes 13 high-quality research articles collected over a period of almost 3 years, with more than 70 authors from different institutions contributing to their scientific outputs.

Wang et al. [1] explored the efficacy of cementitious capillary crystalline waterproofing materials for concrete structure repair, demonstrating a healing rate exceeding 90% under water pressure. Complementary to this, Wang et al. [2] investigated microbial-induced calcium carbonate precipitation (MICP) using Bacillus pasteurii, reporting compressive strength improvements in repaired concrete of up to 11.4%, alongside reduced water permeability. These studies underscore the potential of autonomous healing mechanisms to extend the service life of concrete structures while minimizing maintenance costs.

The integration of fiber-reinforced composites has further expanded the boundaries of material performance. Han et al. [3] examined polyvinyl alcohol (PVA) fiber-reinforced engineered cementitious composites (ECCs) using seawater and coral sand, which achieved a compressive strength of 45.88 MPa and demonstrated exceptional strain-hardening behavior. Such materials not only address brittleness, but also align with sustainable practices by utilizing marine resources. Similarly, Li et al. [4] evaluated ECCs under combined salt frost and sustained flexural loads, revealing that crack widths remained below 100 μm even at 50% deflection levels, highlighting their suitability for infrastructures in harsh environments like the coast. Bu et al. [5] advanced our understanding of chloride transport in cracked ECCs, developing a time- and crack width-dependent model. Their findings revealed that crack widths exceeding 0.2 mm significantly accelerate chloride ingress, with the time-dependent constant n decreasing linearly with crack width. This model provides critical insights for designing ECC structures in marine environments, ensuring long-term durability through controlled crack widths. Zhang et al. [6] further investigated ECCs under coupled sustained flexural loads and accelerated carbonation, demonstrating that even under high stress levels (60% flexural strength), fiber bridging effectively limits crack propagation. Carbonation refined pore structures, enhancing microhardness by 20–30% in carbonated zones, underscoring ECC’s resilience under combined mechanical and environmental stressors.

The role of chemical additives in modulating material properties is another critical topic of research. Shvetsova et al. [7] investigated sodium citrate’s dual functionality as an accelerator and retarder in blended Portland–calcium aluminate cement systems, observing enhanced early strength development and delayed silicate hydration. Lamari et al. [8] explored silane-based admixtures as partial replacements for superplasticizers, noting that methacryloxypropyltrimethoxysilane (MCPTMS) was more effective in preserving workability while reducing compressive strength losses compared to traditional formulations. These findings emphasize the importance of tailored additive strategies to balance processing requirements and end-use performance. Zhou et al. [9] conducted a study on the dispersion optimization and performance of graphene nanoplatelets (GNPs) in cement mortar. An optimal dispersion method combining 10 mg/mL PVP, 15 min high-speed shear (8000 rpm), and ultrasonication significantly enhanced GNPs dispersion in cement mortar, with a 1.0 wt% GNP dosage achieving optimal pressure sensitivity (5.8% resistance change) for structural health monitoring, while 0.3 wt% improved mechanical strength by balancing agglomeration and interfacial bonding.

Li et al. [10] pioneered the use of industrial byproducts—lime mud (LM) and fly ash (FA)—in low-cement foamed concrete for road embankments. By replacing 50–80% of the cement with LM-FA blends, they achieved compressive strengths exceeding 0.8 MPa and California Bearing Ratios (CBR) over 8%, meeting road engineering standards. The high alkalinity of LM accelerated FA hydration, enhancing early strength by 15–20% while reducing CO₂ emissions and landfill waste. This work exemplifies the circular economy’s potential in construction, highlighting the possibility of turning industrial waste into high-value infrastructure materials. The durability of materials under chemical and mechanical stressors remains of paramount importance. Omikrine Metalssi et al. [11] systematically evaluated mortar performance under external sulfate attack (ESA), revealing that lower water-to-cement ratios (0.45) significantly mitigated expansion and strength loss. Kighta et al. [12] developed low-cost TiO₂ composites via ball milling, demonstrating NOx degradation efficiencies comparable to those of high-cost benchmarks like P25, thereby offering an economical solution for air-purifying construction materials. Liu et al. [13] employed the Cohesive Zone Model (CZM) to simulate crack development in tunnel linings with insufficient thickness. Their study revealed that lining thickness reductions of 20–40% altered stress distributions and accelerating crack propagation. This work highlights the criticality of quality control during construction and provides a predictive tool for assessing defect-related risks in aging infrastructure.

As is known, the “Multifunctional Cementitious Composites: Manufacturing and Characterization” is a comprehensive topic, and therefore, it is not possible to include research on all aspects of this field. However, we believe that the papers presented in this Special Issue provide a better understanding of the transformative potential of advanced materials in addressing infrastructure challenges, and we hope that further progress can be achieved based on the ideas introduced in this Special Issue.
